# Generating Gene Ontology-Disease Inferences to Explore Mechanisms of Human Disease at the Comparative Toxicogenomics Database

**DOI:** 10.1371/journal.pone.0155530

**Published:** 2016-05-12

**Authors:** Allan Peter Davis, Thomas C. Wiegers, Benjamin L. King, Jolene Wiegers, Cynthia J. Grondin, Daniela Sciaky, Robin J. Johnson, Carolyn J. Mattingly

**Affiliations:** 1 Department of Biological Sciences, North Carolina State University, Raleigh, North Carolina, United States of America; 2 Department of Bioinformatics, Mount Desert Island Biological Laboratory, Salisbury Cove, Maine, United States of America; 3 Center for Human Health and the Environment, North Carolina State University, Raleigh, North Carolina, United States of America; University of the Sunshine Coast, AUSTRALIA

## Abstract

Strategies for discovering common molecular events among disparate diseases hold promise for improving understanding of disease etiology and expanding treatment options. One technique is to leverage curated datasets found in the public domain. The Comparative Toxicogenomics Database (CTD; http://ctdbase.org/) manually curates chemical-gene, chemical-disease, and gene-disease interactions from the scientific literature. The use of official gene symbols in CTD interactions enables this information to be combined with the Gene Ontology (GO) file from NCBI Gene. By integrating these GO-gene annotations with CTD’s gene-disease dataset, we produce 753,000 inferences between 15,700 GO terms and 4,200 diseases, providing opportunities to explore presumptive molecular underpinnings of diseases and identify biological similarities. Through a variety of applications, we demonstrate the utility of this novel resource. As a proof-of-concept, we first analyze known repositioned drugs (e.g., raloxifene and sildenafil) and see that their target diseases have a greater degree of similarity when comparing GO terms vs. genes. Next, a computational analysis predicts seemingly non-intuitive diseases (e.g., stomach ulcers and atherosclerosis) as being similar to bipolar disorder, and these are validated in the literature as reported co-diseases. Additionally, we leverage other CTD content to develop testable hypotheses about thalidomide-gene networks to treat seemingly disparate diseases. Finally, we illustrate how CTD tools can rank a series of drugs as potential candidates for repositioning against B-cell chronic lymphocytic leukemia and predict cisplatin and the small molecule inhibitor JQ1 as lead compounds. The CTD dataset is freely available for users to navigate pathologies within the context of extensive biological processes, molecular functions, and cellular components conferred by GO. This inference set should aid researchers, bioinformaticists, and pharmaceutical drug makers in finding commonalities in disease mechanisms, which in turn could help identify new therapeutics, new indications for existing pharmaceuticals, potential disease comorbidities, and alerts for side effects.

## Introduction

Manual curation of the scientific literature helps standardize, harmonize, and organize disparate data into a structured format, making it more manageable and computable for analysis [1–2]. Biocurators for the Comparative Toxicogenomics Database (CTD; http://ctdbase.org/) review environmental health and other peer-reviewed literature and manually code a core set of chemical-gene, chemical-disease, and gene-disease interactions using controlled vocabularies and structured notation [3–5]. In 2013, CTD collaborated with Pfizer scientists to manually curate 88,000 articles for interactions between 1,500 therapeutic drugs and their diseases [6]. This collaboration enhanced the scope of CTD information beyond environmental chemicals, and highlighted the goal of understanding chemical toxicity for both environmental health scientists and pharmaceutical drug developers.

To great effect, CTD has utilized data integration to transfer knowledge [7] and generate predictive inferences between different types of curated data [8–9]: if chemical A interacts with gene B, and independently gene B is associated with disease C, then chemical A can be inferred to have a relationship with disease C (via gene B). Integrating CTD’s three core data types (chemical-gene, chemical-disease, and gene-disease) yields chemical-gene-disease inferences that can be statistically evaluated and ranked [10]. This method of knowledge transfer can be used for any type of data, including Gene Ontology (GO) annotations.

The GO is an independent annotation resource of controlled vocabularies used by biocurators to characterize a gene product’s molecular function (GO-MF), cellular component (GO-CC), and biological process (GO-BP) [11]. While CTD biocurators do not annotate genes with GO terms, each month CTD imports the official file of updated GO-gene annotations from NCBI Gene [12] and displays them on “GO” data-tabs for each CTD Gene page as well as on dedicated CTD GO page. These imported GO-gene annotations help describe the functions, processes, and localizations of genes associated with chemicals and diseases in CTD.

GO annotations can also be used for data integration. Previously, we integrated GO-gene annotations with CTD’s gene-chemical interactions to yield GO-chemical inferences [13]. Here, we describe the value of integrating GO-gene annotations with CTD’s curated gene-disease data (in a chemical-independent manner) to produce novel GO-disease inferences. Thus, if gene A is annotated to biological process B (by a GO biocurator), and gene A is independently curated to disease C (by a CTD biocurator), then integration of these two datasets generates an inferred relationship between biological process B and disease C (via gene A). These inferences provide a unique way to compare diseases, since they expand beyond analyzing gene sets, and instead cast a wider net by comparing broader biological concepts like activities and processes. We provide several examples of how these data can be used by investigators for insight into understanding and comparing disease mechanisms, disease predictions, and possible therapeutic repositioning. Over 753,000 inferences connecting 15,700 GO terms to 4,200 diseases are now freely available through the CTD web site.

## Materials and Methods

### CTD’s GO-disease data file

CTD is updated monthly (http://ctdbase.org/about/dataStatus.go). Analysis was derived from data available in CTD in October 2015 (public web application version 14384). Each month, CTD imports and integrates GO-gene annotations from the NCBI using the gene2go file. All Eumetazoa-based species annotations, citing specific evidence codes, and associated with genes included in CTD’s subset of NCBI Gene [12], are incorporated into CTD. GO-disease inferences are generated via shared gene sets between these direct GO-gene annotations from NCBI and CTD’s directly curated gene-disease relationships. CTD’s GO-disease inferences are freely available as downloadable files (http://ctdbase.org/downloads/#godiseasegenes). Prior to subsequent analysis, files were processed to remove three GO parent term placeholders used by external databases: “molecular_function” (GO:0003674), “biological_process” (GO:0008150), and “cellular_component” (GO:0005575).

### CTD analysis tools

CTD provides a suite of web-based, user-friendly analysis tools (http://ctdbase.org/tools/). This report uses: *MyVenn* (http://ctdbase.org/tools/myVenn.go); *VennViewer* (http://ctdbase.org/tools/vennViewer.go); *Batch Query* (http://ctdbase.org/tools/batchQuery.go); and *Set Analyzer* (http://ctdbase.org/tools/analyzer.go). All tools are freely available, and have been described previously [4,13].

### Inferred GO-CC analysis

GO-CC terms were queried using CTD to collect diseases for each specific GO-CC term as well as to any descendent GO-CC term. The collection was filtered to generate unique lists of diseases. GO-CC query terms included: “nucleus” (GO:0005634), “mitochondrion” (GO:0005739), “endoplasmic reticulum” (GO:0005783), “Golgi apparatus” (GO:0005794), and “plasma membrane protein complex” (GO:0098797). The 1,178 diseases associated with mitochondrion were binned into 37 generic disease categories using CTD’s MEDIC disease vocabulary slim list [5].

### Inferred GO-MF analysis

The “GO-Disease molecular function associations” file was sorted to collect all inferred GO-MF terms associated with the top six neoplasms described in the Results: prostate, breast, stomach, lung, hepatocellular carcinoma, and colorectal. CTD’s *MyVenn* tool was used to find the inferred GO-MF subset common to all six cancers. Genes directly associated with the neoplasms were compared using CTD’s *VennVeiwer* tool to discover six genes in common. The 86 GO-MF terms annotated to these six genes were collected using CTD’s *Batch Query* tool.

### Inferred GO-BP analysis

Historical information for the selected repositioned pharmaceuticals was collected from Drugs@FDA (http://www.accessdata.fda.gov/scripts/cder/drugsatfda/index.cfm). CTD’s “GO-Disease biological process associations” file was sorted to collect GO-BP terms inferred to disease targets for each repositioned drug. Since CTD’s MEDIC disease vocabulary is hierarchical [5], we collected data curated to the parent disease term plus data curated to any descendent (i.e., disease sub-type). For example, in the analysis of raloxifene, we combined GO-BP terms and genes directly annotated to “osteoporosis” (MESH:D010024) as well as disease sub-types, such as “osteoporosis, postmenopausal” (MESH:D015663). This insured the most accurate comparison for drug targets. A complete list of the diseases, inferred GO-BP terms, and direct genes used for these analyses is provided in S1 File. The disease-pair matrix was created for the 4,258 diseases with 10,640 GO-BP inferences to compare the number and profile of genes and inferred GO-BP terms shared between any two pairs of diseases (A and B). The matrix was created computationally using a process to extract all curated disease-gene associations from CTD’s “Gene-disease associations” file (http://ctdbase.org/reports/CTD_genes_diseases.tsv.gz) and all GO-BP term-disease inferences from CTD’s “GO-Disease biological processes associations” file (http://ctdbase.org/reports/CTD_Disease-GO_biological_process_associations.tsv.gz). Each disease with one or more inferred GO-BP terms was compared to every other disease to compute the number of associated genes (for each disease A and B), the intersection of associated genes (between disease A and B), the number of associated GO-BP terms (for each disease A and B), and the intersection of associated GO-BP terms (between disease A and B). The matrix compilation was loaded to a PostgreSQL database for further manipulation. SQL-based queries were run against this database to retrieve data described in the text. Similarity indices were computed for disease-pairs involving bipolar disorder using the Jaccard method, as previously described [14]. This index was calculated as the number of intersecting inferred GO-BP terms for diseases A and B divided by the union of inferred terms for A and B. We compared the top 20 ranked results against the DiseaseComps publicly available in CTD for bipolar disorder via shared gene associations.

### Statistics

For repositioned pharmaceuticals, the significance of overlap among inferred GO-BP terms for disease-pairs were evaluated by computing 2x2 contingency tables and applying the hypergeometric distribution in R 3.2.1 (http://www.r-project.org). For the three diseases influenced by lithium, Pearson’s Chi-square test in R was used to determine the significance of overlap among inferred GO-BP terms for the three diseases. Contingency tables are provided in S2 File.

## Results and Discussion

### Inferred GO-disease relationships from CTD

**Fig 1:**
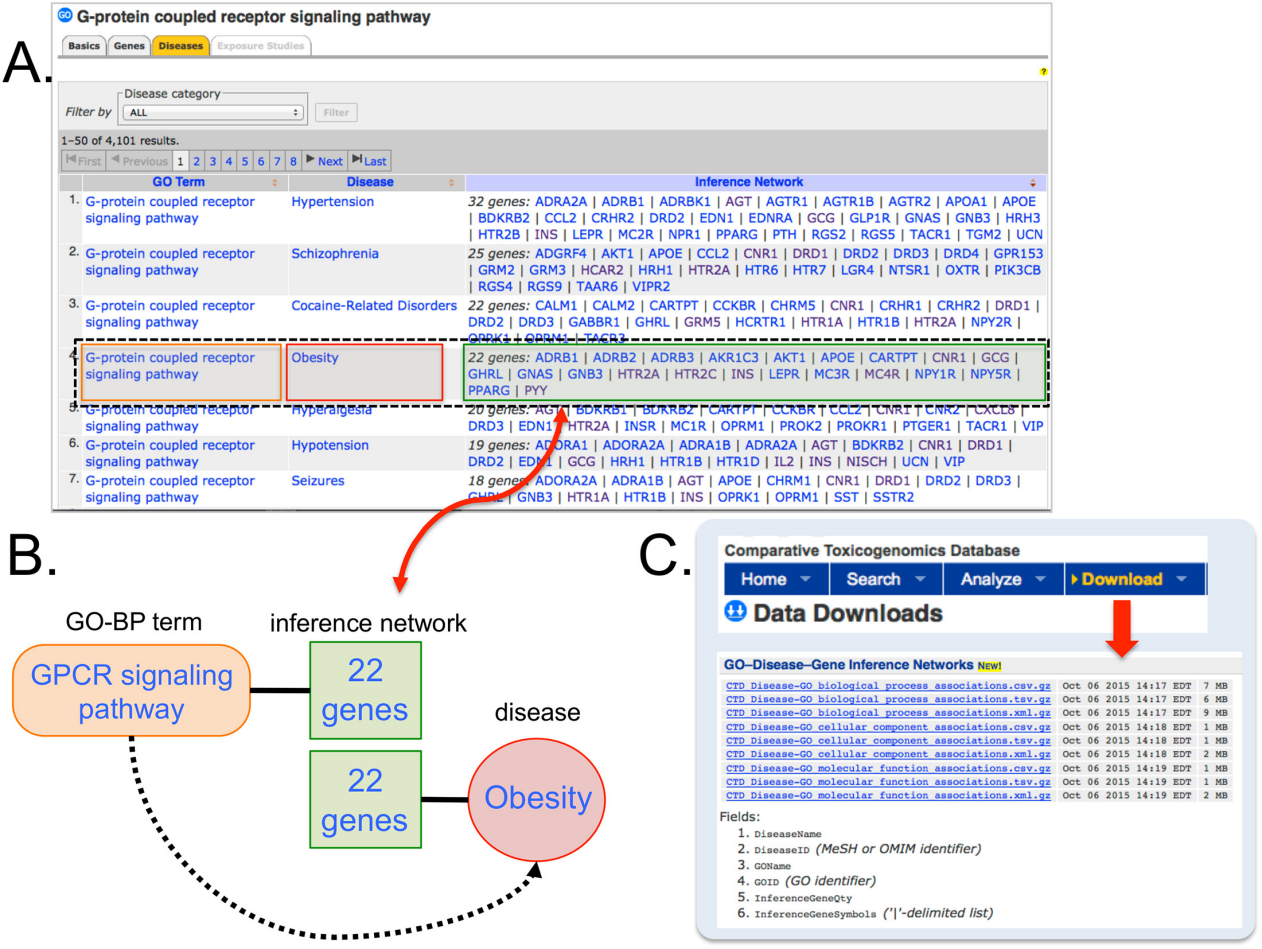
GO terms inferred to diseases via gene inference networks. (A) The "Diseases" data-tab on CTD’s webpage for the GO-BP term “G-protein coupled receptor signaling pathway” lists human pathologies inferred to this GO term, including a connection to obesity made by an inference network of 22 genes (red double arrow). (B) A schematic outlines how this GO term is directly annotated to these 22 genes (by external databases) which, in turn, have also been directly associated with obesity independently by CTD biocurators from the literature, allowing the GO term to be inferred (dotted black arrow) to the disease. (C) The files for "GO-Disease-Gene Inference Networks" are freely available from CTD's "Data Downloads" page and can be retrieved in a variety of formats.

CTD contains 32,260 directly curated gene-disease interactions between 7,950 genes and 4,958 diseases. Additionally, CTD imports and displays 1,155,024 gene-GO annotations from NCBI Gene [12]. GO-disease inferences are computationally generated at CTD through data integration (Fig 1A and 1B). The inferences can be viewed on the “Diseases” data-tab at any GO page in CTD (http://ctdbase.org/voc.go?type=go). Since GO is a hierarchy, disease inferences are subsumed to parent terms; for example, the GO term “apoptotic process” (GO:0006915) displays all diseases inferred to that specific GO term as well as to descendents of that term, such as “activation-induced cell death of T cells” (GO:0006924). This structure allows users to easily find all data associated with any GO domain as well as drill down to more granular GO terms to refine the specificity and associated data retrieved. We have now formatted all of these computed GO-disease inferences into structured files that are freely available from the “Data Downloads” page (http://ctdbase.org/downloads/) for the three branches: GO-CC, GO-MF, and GO-BP (Fig 1C). Currently, the files contain 753,346 inferences between 15,707 GO terms and 4,277 diseases, inferred by 6,766 genes (Table 1). GO-BP has the greatest number of associated inferences since, on average, genes tend to be annotated with more GO-BP than GO-MF or GO-CC terms. The top 10 diseases with the greatest number of inferred GO terms include six cancers, autism, hypertension, glomerulonephritis, and peripheral nervous system diseases (Table 2).

**Table 1:** CTD data content for GO terms inferred to diseases.

Inference set	No. inferences	No. GO terms	No. diseases	No. genes
GO-BP to disease	539,120	10,640	4,258	6,570
GO-MF to disease	123,680	3,753	4,187	6,320
GO-CC to disease	90,546	1,314	4,254	6,500
Total	753,346	15,707	4,277	6,766

**Table 2:** Diseases with the greatest number of inferred GO terms (via number of genes).

Disease	No. inferred GO-BP terms (via genes)	No. inferred GO-CC terms (via genes)	No. inferred GO-MF terms (via genes)	Total (via genes)
Prostatic neoplasm	4,142 (468)	493 (479)	1,136 (465)	5,771 (482)
Breast neoplasm	4,010 (411)	403 (396)	930 (398)	5,343 (420)
Stomach neoplasm	3,103 (272)	386 (278)	731 (273)	4,220 (279)
Lung neoplasm	2,997 (178)	284 (174)	679 (172)	3,960 (178)
Hepatocellular carcinoma	2,854 (217)	311 (217)	805 (215)	3,970 (219)
Autistic disorder	2,805 (234)	317 (232)	641 (226)	3,763 (235)
IGA glomerulonephritis	2,752 (411)	429 (425)	983 (409)	4,164 (432)
Hypertension	2,752 (177)	265 (177)	636 (177)	3,653 (177)
Colorectal neoplasm	2,733 (219)	315 (216)	671 (213)	3,719 (220)
Peripheral nervous system diseases	2,696 (276)	344 (277)	717 (274)	3,757 (283)

Below we demonstrate some of the many ways investigators can utilize this free resource to explore and address disease mechanisms from the perspectives of cellular components, molecular functions, and biological processes.

### Exploring diseases from GO-CC perspective

**Fig 2:**
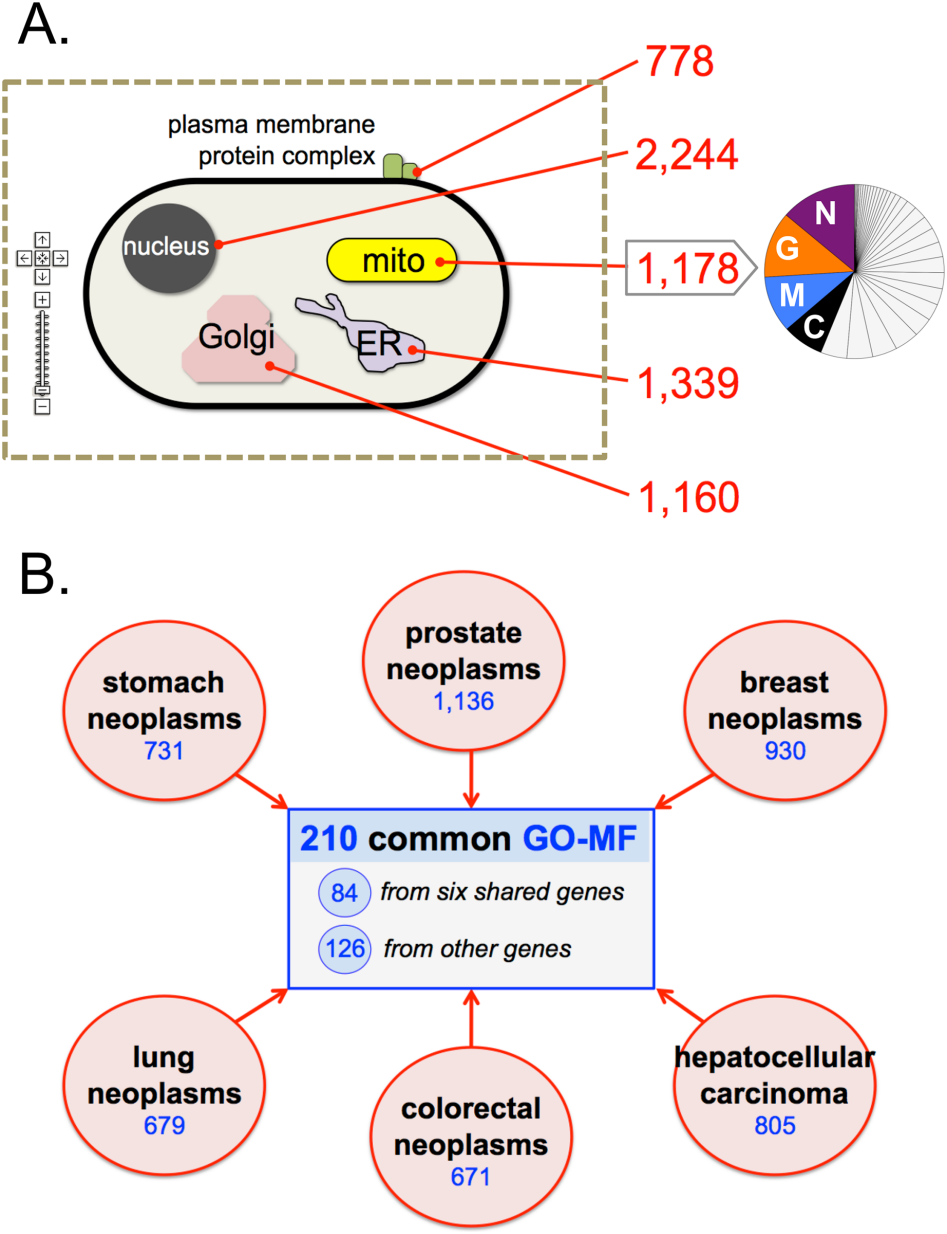
Exploring disease mechanisms from a GO perspective. (A) Using inferred GO-CC data, the number of diseases (red numbers) can be associated with cellular locations, providing an additional level of information for potential, druggable targets. Interactive cell maps can be annotated with these inferences to allow navigation and exploration. The 1,178 diseases mapping to the mitochondrion (boxed arrow) were clustered to MEDIC disease categories (pie chart), and the top four categories are highlighted: nervous system diseases (N), genetic inborn diseases (G), metabolic diseases (M), and cancers (C). (B) The inferred GO-MF terms (blue numbers) for six cancers (red circles) share a subset of 210 molecular functions (blue box), providing core molecular activities informing common mechanisms of cancer.

Based upon inferred GO-CC terms in CTD, diseases can be mapped to cellular locations (Fig 2A). This unconventional way of presenting and exploring pathologies can uniquely pinpoint interesting features, such as the 778 diseases inferred to protein complexes on the plasma membrane, which could have implications on drug targeting. The 1,178 diseases related to the mitochondrion can help expand and inform the compendium of known mitochondrial pathologies [15]. Towards that end, we classified the 1,178 mitochondrial-associated disorders into generic disease categories using CTD’s MEDIC ‘slim list’ [16] to reveal the types of diseases that map to this organelle (Fig 2A). Ranked the most abundant (14% total) were nervous system diseases, a recognized pathology of several mitochondrial defects [17], followed by genetic inborn diseases (12% total) and metabolic diseases (10% total), which are consistent with the mitochondrion being a maternally inherited metabolic workhorse of the cell; cancers are ranked fourth (8% total), providing information that could be helpful for expanding the field of mitochondrion-targeted cancer therapies [18].

As well, users can leverage CTD’s GO-CC inferences to develop new visualization tools. For example, a schematic cell populated with CTD’s inferred GO-CC localizations would produce a scalable atlas that allows scientists to take a virtual tour of the cell and explore the disease landscape in unprecedented ways. Zooming features would allow users to see annotations at specific intracellular sites, such as the 13 diseases currently inferred to the specific “ER-mitochondrion membrane contact site” (GO:0044233) or the nine diseases associated with “RNA polymerase II transcription repressor complex” (GO: 0090571) found within the nucleus.

### Exploring diseases from GO-MF perspective

One potential untapped use of CTD information is in the burgeoning field of drug repositioning (or repurposing): the process of finding a new therapeutic use for a previously tested or approved pharmaceutical [19]. A variety of different bioinformatics and computational approaches have been adopted and merged to identify candidates for drug repositioning, including gene expression arrays, chemical structure similarities, and protein-protein interaction maps [20–24].

CTD’s inferred GO terms that are shared between different diseases are a type of ‘big data’ [25] that also could be exploited in this endeavor. For example, analysis of the inferred GO-MF terms associated with the top six cancers (Table 2) reveals a shared subset of 210 molecular functions (Fig 2B). This commonality is not entirely due simply to overlapping genes. In fact, only six genes (CCND1, EGFR, PIK3CA, PTGS2, TP53, and TYMS) are common to all six cancers, accounting for 84 of the GO-MF terms (40%), whereas the remaining 126 shared GO-MF terms (60%) derive from discrete disease-specific gene sets. Of these 126 common terms, only 15 (12%) describe broad, generic functions (as defined by their low granularity level positions of 1 or 2 in the GO hierarchy; S3 File), such as “catalytic activity” (GO:0003824; level 1) and “oxygen binding” (GO:0019825; level 2). The remaining GO terms, however, are more granular and include 22 terms (17%) at GO level 3, 39 terms (31%) at level 4, and 50 terms (40%) at the higher levels 5 through 9 (S3 File). These more specific terms may help to identify targetable molecular functions, such as “ubiquitin protein ligase activity” (GO:0061630; level 5), “serine-type endopeptidase inhibitor activity” (GO:0004867; level 6), and “androgen receptor binding” (GO:0050681; level 7). Such an analysis suggests that strict reliance on common genes when comparing diseases might belie biological similarity that exists at the functional level.

### Exploring diseases from GO-BP perspective: proof-of-concept

Comparing the inferred GO-BP terms (rather than individual gene sets) associated with two diseases casts a wider net to identify overlaps between shared biological processes. This information can also be used to detect previously unrecognized commonalities between disparate diseases to discover potential new disease targets for existing pharmaceuticals, identify possible comorbidities for known diseases, and discern potential side effects for therapeutics.

**Fig 3:**
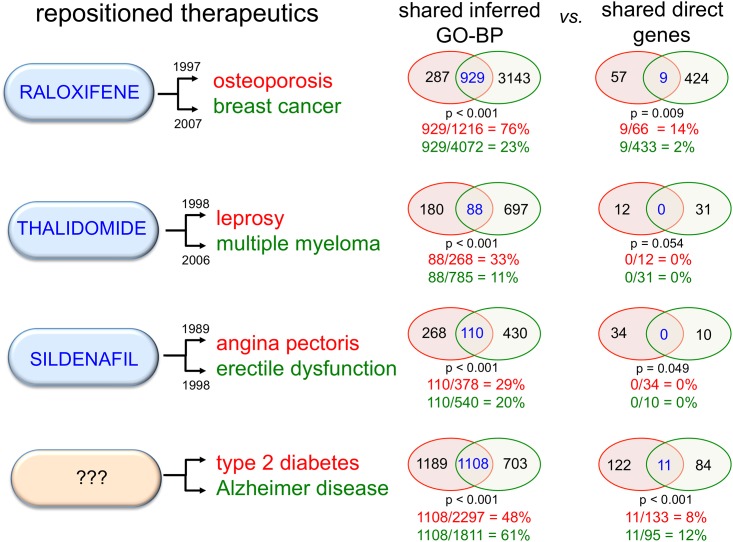
The potential for shared GO-BP terms vs. shared genes to better inform the repositioning of pharmaceuticals. Three repositioned therapeutics are shown (blue ovals) with their initial disease target (red) and their subsequent new indication (green), with FDA approval/patent dates listed. The fourth example (orange oval) is purely hypothetical for a presumptive therapeutic that treats both type 2 diabetes and Alzheimer disease, based upon the extensive amount of shared GO-BP terms. Venn diagrams show there is a greater amount of overlap for inferred GO-BP versus directly curated genes for the disease-pairs for each drug, including two therapeutics (thalidomide and sildenafil) for disease-pairs that do not share any genes, but do share inferred GO-BP terms. Venn circles and percentages are color-coded to match targeted diseases in each example; significance of overlaps is defined by p-values.

As a proof-of-concept, we performed a side-by-side comparison of shared inferred GO-BP terms vs. shared genes for the disease targets of three well-known, repositioned therapeutics: raloxifene, thalidomide, and sildenafil (Fig 3). For all three cases, the percent overlap of inferred GO-BP terms was greater than that for their shared genes.

Raloxifene was originally used to treat types of osteoporosis [26], but it has since been successfully used for treatment of specific incidences of invasive breast cancer [27]. These diseases share only nine genes, but have a total of 929 inferred GO-BP terms in common. Of these GO-BP terms, 500 (54%) derive from the shared genes, whereas the remaining 429 GO-BP terms (46%) derive from distinct genes. Osteoporosis and breast cancer appear to have a higher degree of biological similarity based on common biological processes vs. individual genes (Fig 3).

Sildenafil, originally developed as an anti-angina therapeutic by Pfizer, had the surprising side effect of penile enlargement in volunteers during phase 1 clinical trials, and has since been successfully re-marketed as Viagra for erectile dysfunction [28]. Thalidomide was initially used as a sedative for pregnant mothers in the late 1950s, but had disastrous teratogenic consequences to developing babies and was quickly removed from the market [29]. However, in 1965 it was serendipitously found to improve leprosy [30], and now its anti-angiogenesis properties [31] are recognized as a powerful weapon against tumor development, leading to the repositioning of the drug as a successful treatment for several types of cancer, most notably multiple myeloma [32]. Both thalidomide and sildenafil are examples of repositioned pharmaceuticals where their primary and secondary disease targets currently have no common genes in CTD (Fig 3). Interestingly, however, the diseases treated by these pharmaceuticals overlap with respect to inferred GO-BP terms (11–33% for the thalidomide disease targets and 20–29% for sildenafil targets), suggesting that shared processes and pathways (and not necessarily shared genes) can still provide important insight for drug re-evaluation.

Finally, a fourth example supports a potential rationale for common treatments of type 2 diabetes and Alzheimer disease (AD). A compelling epidemiological and mechanistic link (focused on insulin and glucose) between these two seemingly disparate diseases has been recently recognized in the literature [33–35], with some investigators now referring to AD as “type 3 diabetes” [36]. The possibility to treat AD with a repurposed, existing diabetes medication is intriguing. Again, CTD provides data suggesting a greater degree of biological overlap based on common GO-BP terms, as opposed to gene sets, for these two pathologies (Fig 3).

### Exploring diseases from GO-BP perspective: computational generation of disease-pairs

Based on our examples above, we next developed a computational process to identify comparable diseases using shared inferred GO-BP terms. As noted, in CTD there are currently 4,258 diseases with 10,640 inferred GO-BP terms (Table 1). We systematically compared these 4,258 disease terms against each other to calculate the percent overlap for shared curated genes vs. shared inferred GO-BP terms for each disease-pair. This resulted in a matrix of 9,063,154 disease-pair combinations. When analyzed for the greatest number of shared genes and/or inferred GO-BP terms, the top 100 disease-pairs almost exclusively corresponded to cancer-related associations: “adenocarinoma-lung neoplasms”, “breast neoplasms-lung neoplasms”, “neoplasm invasiveness-neoplasm metastasis”, “breast neoplasms-prostate neoplasms”, etc. This is likely due not only to an inherent underlying similarity for these diseases, but also to the fact that cancer is the most directly curated disease in CTD [16], and as more genes are curated to neoplasms, this highly interconnected “cancer network” will continue to grow. While these cancer disease-pairs are still informative (especially with respect to understanding disease etiology and exploring potential new treatment options), we were curious to see what predictions might still emerge from our analysis by testing the system further. Towards that end, we added arbitrary filters to reduce the prevalence of already-known disease connections that were populating the top hits due to overwhelming shared genes (and, consequently, their shared inferred GO terms).

**Fig 4:**
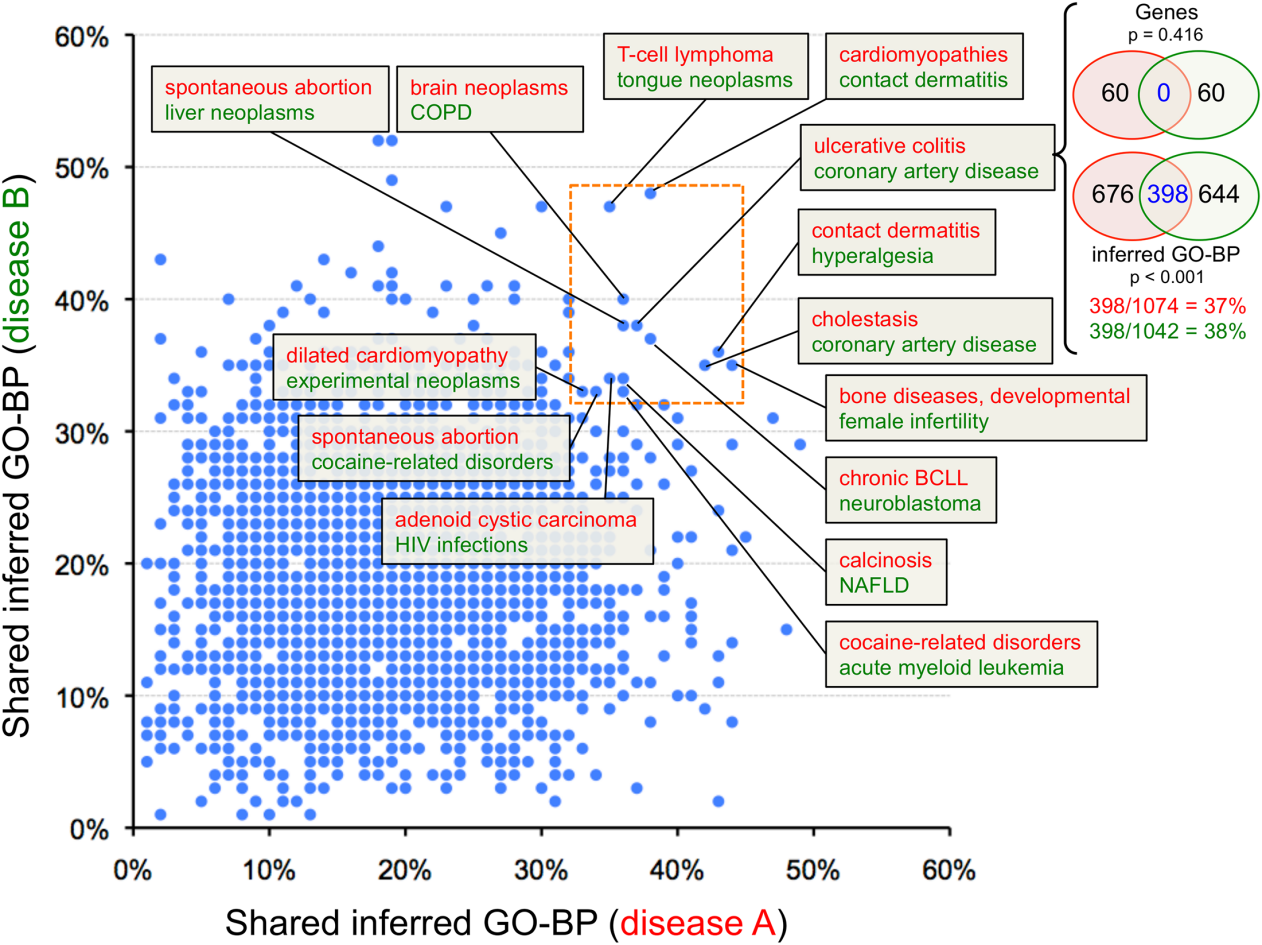
Discovering comparable diseases via shared inferred GO-BP terms. There are 2,457 disease-pairs (blue dots) that do not share any genes, but do share inferred GO-BP terms. The percentage of overlap between inferred GO-BP terms for disease A (red, x-axis) is graphed against those of disease B (green, y-axis) to find heterogeneous diseases that are comparable to each other based exclusively on shared biological processes (and no shared genes). A set of 14 disease-pairs with a high amount of shared overlap for both diseases is indicated (orange dotted box). As an example, ulcerative colitis (disease A, red) has no genes in common with coronary artery disease (disease B, green), but the two share 398 inferred GO-BP terms, graphed as 37% for ulcerative colitis and 38% for coronary artery disease. Disease abbreviations: COPD (chronic obstructive pulmonary disease), BCLL (B-cell lymphocytic leukemia), NAFLD (non-alcoholic fatty liver disease). Note: many disease-pairs have the same coordinates (rounded to 2-digits), and thus appear as only a single dot on the graph.

Two filters were applied to reduce the matrix size. First, to avoid the confounding issue of shared inferred GO-BP terms being due to shared curated genes, we filtered the matrix to only disease-pairs that did not have any curated genes in common. This reduced the matrix to 4,778,760 disease-pairs with one or more inferred GO-BP terms in common. Next, we restricted the dataset to disease-pairs wherein each disease (A and B) had 10 or more directly curated (but discrete) genes, and the number of discrete genes curated to the two diseases had to be within 10% of each other. Thus, if disease A had 50 curated genes associated with it, then disease B had to have 45–55 directly curated (but distinct) genes associated with it. This arbitrary second filter was to help ensure that the two diseases being compared had a similar scope of curated content, and removed comparisons between well-characterized diseases (that might have hundreds of associated genes) with lesser-studied diseases (that might have only a few associated genes). This reduced the matrix to 2,457 disease-pairs (S4 File; distribution analysis in S5 File). The overlap of inferred GO-BP terms for these 2,457 disease-pairs identified a variety of pathologies exclusively on the basis of shared inferred GO-BP terms rather than specific genes using the strict filters outlined above (Fig 4). Some of the disease-pairs with the greatest percentage of inferred GO-BP overlap include “brain neoplasms-chronic obstructive pulmonary disease”, “cardiomyopathies-contact dermatitis”, and “ulcerative colitis-coronary artery disease”, the latter of which has been recently confirmed in the literature [37]. Our method (using the two filters) represents just one of the many diverse ways that investigators can sort CTD’s new “GO-Disease-Gene Inference Network” files to explore mechanisms and functionality to make connections between seemingly heterogeneous pathologies. Other more relaxed filtering strategies might permit genes to be shared between the diseases (to boost the similarity measurement between the disease-pairs), or remove the mandate that there be an arbitrary 10% range between the numbers of genes associated with each curated disease-pair. These could also provide informative, productive results, as seen in the post hoc analysis of the already known repositioned pharmaceuticals (Fig 3). Each investigator should design the most suitable way to interpret, sort, analyze, and explore the disease connections that best fit their research objectives.

**Fig 5:**
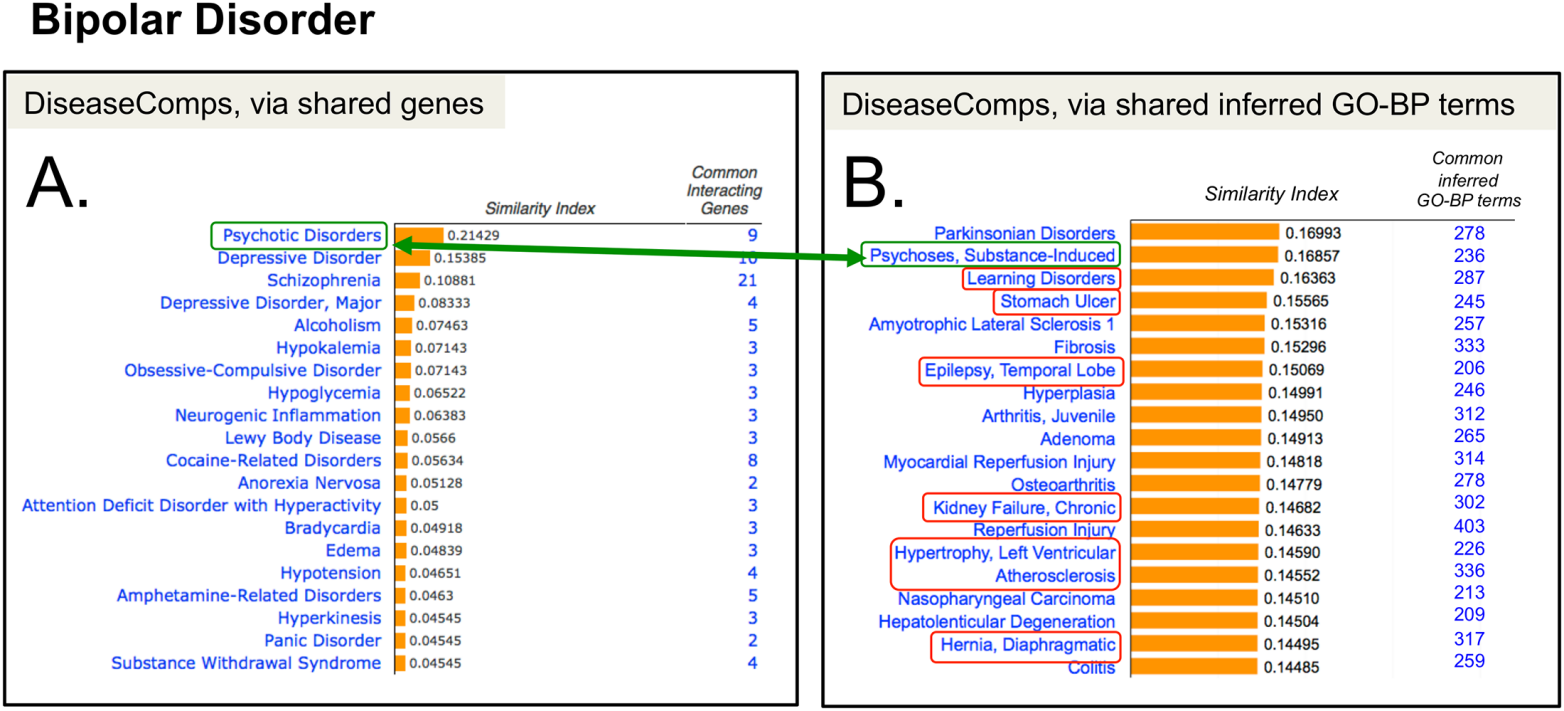
Complementary approaches to discovering comparable diseases. Bipolar disorder is used as a test case to find comparable diseases (DiseaseComps) via two methods. (A) One of CTD’s current methods uses shared genes to compute a statistical similarity index that ranks comparable diseases, and includes psychotic disorders as the top hit for bipolar disorder (green box). (B) An alternative, complementary approach is to use only shared inferred GO-BP terms to find similar diseases that share biological processes (without sharing genes). Here, substance-induced psychoses (green box) is highly scored and redolent of psychotic disorders found using genes (connecting green arrow). Interestingly, other heterogeneous pathologies (red boxes) predicted to be comparable to bipolar disorder have been verified in the recent literature (see text).

Previously, we reported a computational process that ranks comparable diseases (“DiseaseComps”) based upon the number of shared genes using a Jaccard-based similarity index [14]. We employed this same statistical method here to generate similarity indices for ranking our matrix of disease-pairs based upon the number of shared inferred GO-BP terms (in the absence of shared genes). This strategy provides a unique, complementary approach to finding related diseases via the standard method of using shared gene lists. Here, we use bipolar disorder as an example of how this technique provides insights into disease mechanisms. We computed similarity indices for diseases related to bipolar disorder based upon the number of shared inferred GO-BP terms. One of the top comparable diseases (based on 236 shared inferred GO-BP terms and no shared genes) was substance-induced psychoses (Fig 5B), which is redolent of psychotic disorders, the top comparable disease found via 9 shared genes (Fig 5A). Interestingly, many of the other comparable diseases found exclusively using inferred GO-BP terms (Fig 5B) were recently confirmed in the literature as comorbidities or medical conditions in bipolar patients, including learning disorders [38], stomach ulcers [39], epilepsy [40], and atherosclerosis with cardiovascular diseases [41], further validating this approach.

**Fig 6:**
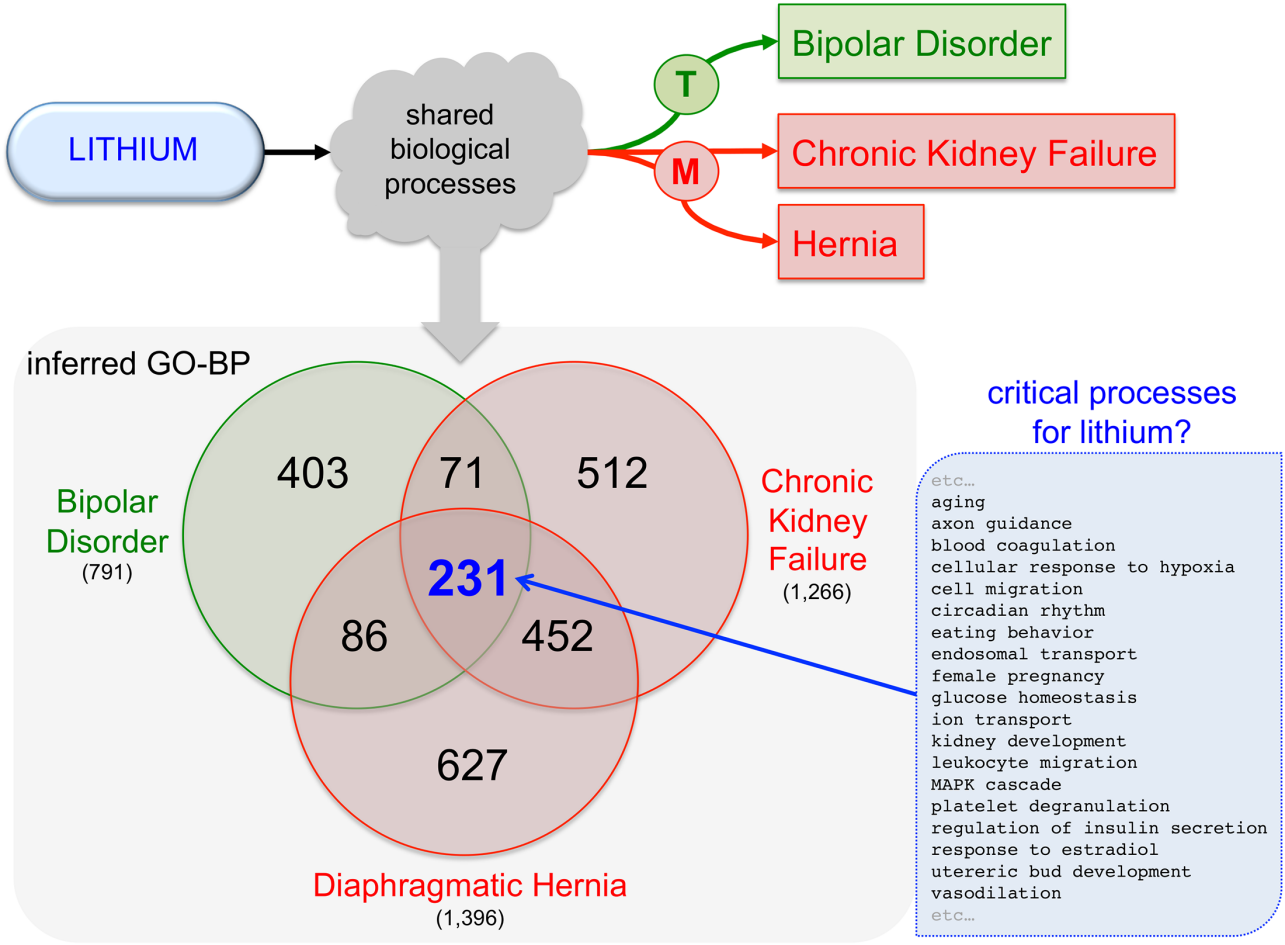
Potential biological processes-of-action for lithium. The drug lithium is a common therapeutic (T) for bipolar disorder (green arrow), but chronic use in patients has also been reported to cause (M) adverse reactions, such as kidney failure and congenital diaphragmatic hernia (red arrows). Assuming the drug works through modulation of biological processes (gray cloud), we used Venn analysis to compare the number of inferred GO-BP for these three outcomes (colored circles). Currently, there are 231 inferred GO-BP terms (p < 0.001) shared that might represent some of the critical biological processes modulated by lithium treatment; a random selection of some of these shared terms is listed (blue subset).

Conserved disease mechanisms might not necessarily translate to shared therapies but may help to explain unintended side effects that share biological mechanisms modulated by a drug. In this regard, the same methods could be equally useful in predicting alerts for potential side effects from target treatment. For example, chronic kidney failure and diaphragmatic hernia were predicted to share similarities with bipolar disorder (Fig 5B). Both of these conditions have been reported as adverse side effects of lithium [42–43], the most common treatment for bipolar disorder. Analysis of shared inferred GO-BP terms for these three seemingly heterogeneous diseases identified a subset of 231 common processes (Fig 6). Contextual knowledge of either these common processes or processes unique to these disorders could provide important insight into the mechanisms that would explain why lithium is an effective treatment for bipolar disease, but also leads to adverse outcomes of kidney failure or hernia.

### Leveraging additional CTD content to generate testable hypotheses

Investigators can use GO-disease inferences in conjunction with CTD curated content in a variety of applications to construct testable hypotheses about the molecular mechanisms of diseases.

**Fig 7:**
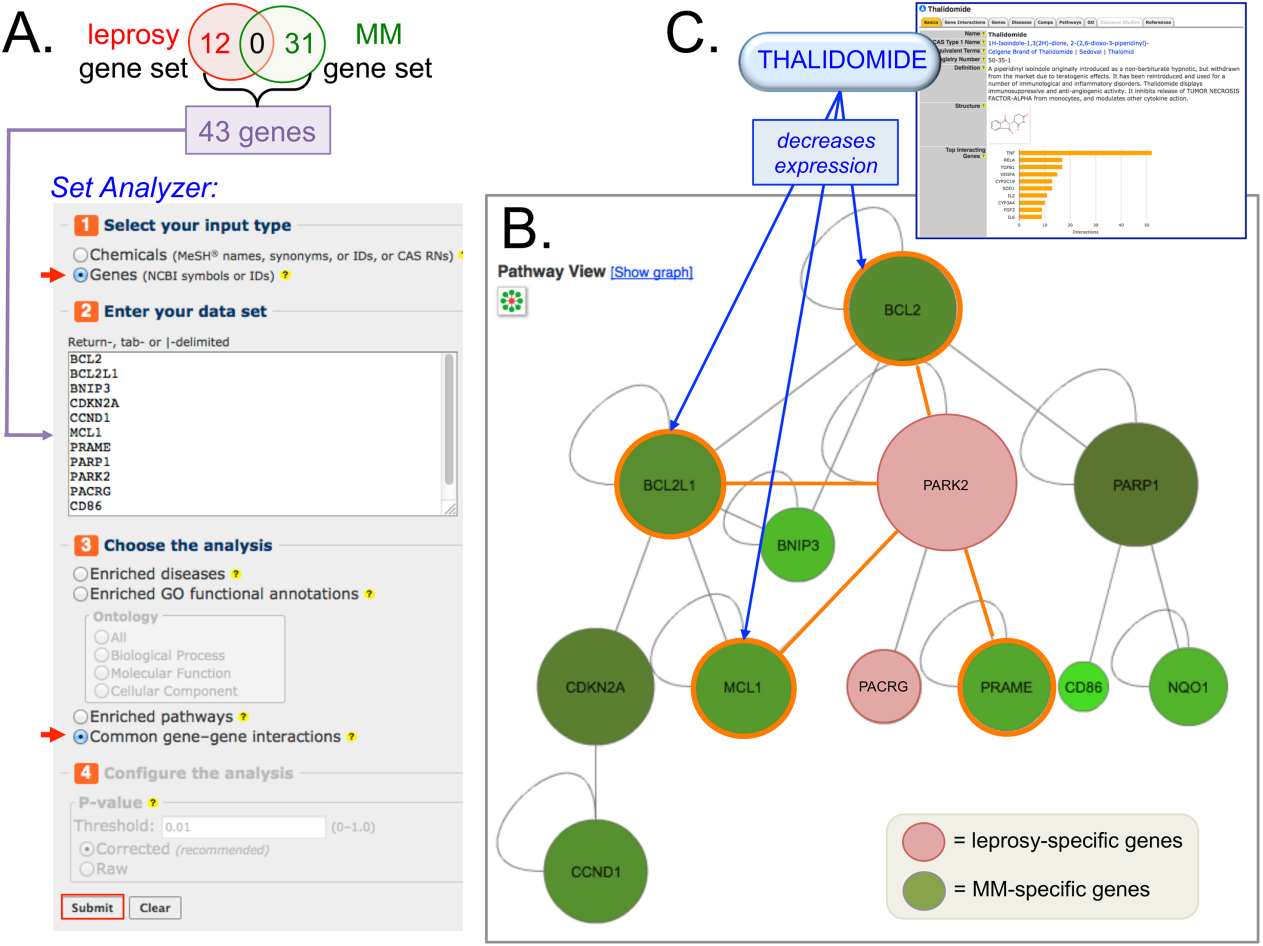
Leveraging CTD content to build a molecular nexus. (**A**) Leprosy and multiple myeloma (MM) are both treated by the drug thalidomide, but the diseases do not currently share any genes in CTD. CTD’s *Set Analyzer* tool can be used to determine whether the disease-specific gene sets function in a common pathway by: selecting “Genes” (top arrowhead), entering the non-overlapping 43 gene symbols for the two diseases, and then selecting “common gene-gene interactions” (bottom arrowhead). (**B**) The resulting interaction network can be customized as a graph using the “Pathway View” icon; genes are displayed as circles and their genetic interactions are represented as gray lines. Here, the graph reveals that one leprosy-specific gene (PARK2; red circle) physically interacts with four MM-specific genes (BCL2, BCL2L1, MCL1, and PRAME; green circles with orange borders). Note: for simplicity, only the relevant genes are shown in the interaction network. (**C**) Leveraging the curated chemical-gene interactions found on CTD’s page for “Thalidomide” (upper right-hand screenshot) reveals that the drug decreases the expression (blue arrows) of three of the genes (BCL2, BCL2L1, and MCL1) that interact with the leprosy-specific PARK2.

First, CTD content can elucidate how a drug might treat two seemingly unrelated diseases (with no shared genes but shared inferred GO-BP terms). For example, thalidomide is used to treat both leprosy and multiple myeloma. Although these two diseases do not share any genes in CTD, they do have many shared inferred GO-BP terms (Fig 3), suggesting a potential common underlying mechanism. There are several possible explanations as to how a chemical could affect two diseases that have no shared genes. The most obvious is a ‘knowledge gap’, where the complete set of genes involved in both diseases is not yet known. Other possibilities are that the drug might target multiple gene products, or that the disease-specific gene sets might overlap in a molecular network that can be modulated by the same chemical. To test the latter possibility, we analyzed the 12 genes associated with leprosy (S1 File) and the 31 genes associated with multiple myeloma (S1 File) using CTD’s *Set Analyzer* tool to look for common gene-gene interactions shared between the two sets (Fig 7A). Leveraging this additional information in CTD revealed that one of the leprosy-associated genes (PARK2) physically interacts with four genes associated with multiple myeloma (BCL2, BCL2L1, MCL1, and PRAME), providing a potential molecular nexus through which thalidomide could act on both diseases (Fig 7B). Furthermore, the curated chemical-gene interactions for thalidomide in CTD (Fig 7C) indicate that this drug decreases the expression of three of these genes (BCL2, BCL2L1, and MCL1), providing a testable hypothesis as to how one chemical could influence two diseases that currently share no genes but do share inferred GO-BP terms.

**Fig 8:**
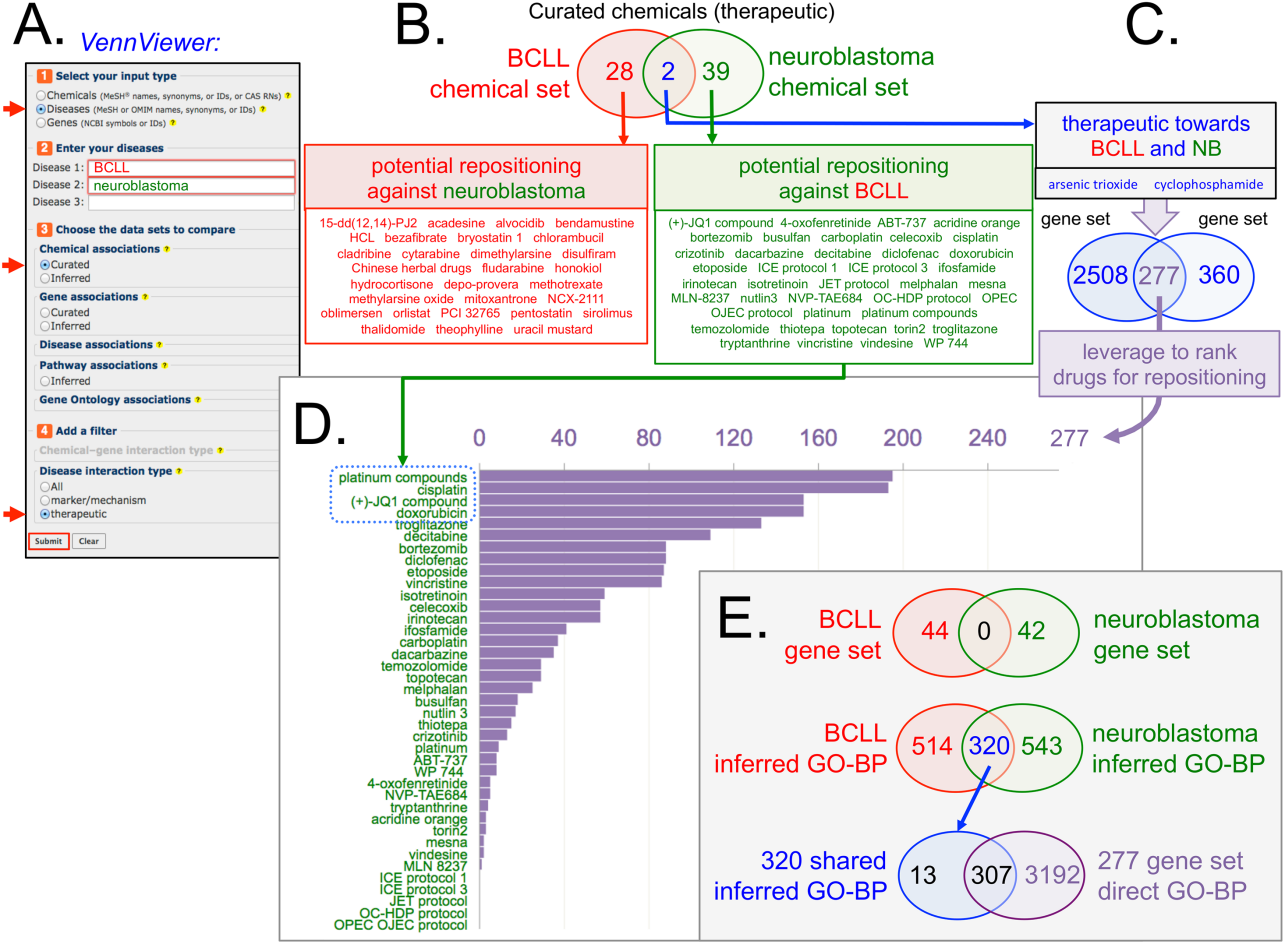
Leveraging CTD content to prioritize drugs for repositioning. B-cell chronic lymphocytic leukemia (BCLL) and neuroblastoma are diseases that currently do not share any known genes in CTD, but do share 320 inferred GO-BP terms, suggesting molecular similarity (see Fig 4). (**A**) Diseases can be compared using CTD’s *VennViewer* tool by selecting “Disease” analysis (top arrowhead), inputting the two disease terms, choosing to compare curated chemical associations (middle arrowhead), and adding a filter to retrieve only therapeutic interactions (bottom arrowhead). (**B**) The resulting Venn diagram identified two chemicals (arsenic trioxide and cyclophosphamide) that each have a curated therapeutic relationship with both diseases, as well as 28 chemicals specific to BCLL (which could potentially be repositioned for neuroblastoma; red box), and 39 chemicals specific to neuroblastoma (which could now be repositioned for BCLL; green box) (**C**) Arsenic trioxide and cyclophosphamide treat both diseases and both chemicals interact with a set of 277 genes (blue Venn circles), information which can be leveraged to help rank the test drugs. (**D**) The 39 therapeutic drugs for neuroblastoma with potential repositioning towards BCLL (green names on y-axis) were queried in CTD to see how many of the 277 genes interact with each test drug (x-axis). Four of the 39 test drugs interact with more than 50% of the 277 genes (blue dotted box). (**E**) Venn diagrams summarize how BCLL and neuroblastoma do not currently share any genes in CTD, but do share 320 inferred GO-BP terms (based upon CTD’s new GO-Disease inference dataset), and that 307 of these 320 GO-BP terms are annotated to the 277-gene set used to rank the test drugs for potential repositioning.

Second, CTD content can help inform the process of drug repositioning by detecting common molecular events. As an example, one of the top disease-pairs from Fig 4 is “B-cell chronic lymphocytic leukemia (BCLL)–neuroblastoma”. These two diseases do not share any genes, but do overlap with 320 inferred GO-BP terms (Fig 8E), suggesting a common molecular underpinning between the two different cancers, and supporting the hypothesis that drugs that treat one disease might be reasonable candidates for repositioning as a treatment for the other disease. Using CTD’s *VennViewer* tool, we retrieved the chemicals that have a therapeutic relationship to these diseases (Fig 8A and 8B). Two of the chemicals (arsenic trioxide and cyclophosphamide) were curated from the literature as potential therapies for both diseases in CTD (Fig 8B, Venn intersection, blue arrow). Since BCLL and neuroblastoma significantly overlap with respect to inferred GO-BP terms, the 39 chemicals currently associated with neuroblastoma (Fig 8B, green Venn subset) are potential candidates to also treat BCLL. We leveraged the additional curated content in CTD to help prioritize these 39 chemicals. Using the *VennViewer* tool again, we found that arsenic trioxide interacts with 2,785 genes, cyclophosphamide interacts with 637 genes, and the two chemicals overlap with respect to 277 shared genes with which they both interact (Fig 8C). Assuming that a therapeutic drug works by interacting with gene product(s), then this 277-gene set can be leveraged as a molecular milepost to rank the 39 test drugs based upon the extent of their interactions (Fig 8D). Four of the 39 chemicals interact with more than 50% of the 277 genes, prioritizing them as perhaps better initial candidates. Among this top set are the well-studied platinum-containing cancer drug cisplatin and the novel compound JQ1 (a small molecule inhibitor of bromodomain-containing proteins) that has shown promise against a multitude of other diseases, including cancers [44]. Furthermore, it is interesting to note that 307 of the 320 (96%) inferred GO-BP terms shared between BCLL and neuroblastoma from the outset are also directly annotated to the 277-gene set (Fig 8E).

## Summary and Future Directions

We describe CTD’s new resource of 753,000 inferences between 15,700 GO terms and 4,200 diseases. This novel dataset (freely available as a downloadable file and integrated into our public web application) provides unique insight into identifying common mechanisms of human diseases, potential drug repositioning, side-effect alerts, and putative comorbidities. We demonstrate the utility of this resource with numerous examples.

Inferred GO-CC terms can be used to map disease concepts to cell regions (such as the 778 diseases inferred to targetable plasma membrane complexes and the 1,178 diseases inferred to the mitochondrion), and new visualization strategies can use this information to design interactive maps to explore pathologies from a sub-cellular viewpoint. Using inferred GO-MF terms, we identify 126 shared molecular functions for six common cancers, leading to potential strategies for designing drugs to target multiple tumor types. A computational comparison of inferred GO-BP terms predicts over 2,400 highly similar disease-pairs based exclusively on shared GO terms, many of which we later confirmed in the literature. Leveraging curated content already in CTD, we demonstrate how thalidomide could potentially treat multiple diseases that currently do not share any known genes, but do significantly overlap with inferred GO-BP terms. Finally, we illustrate how CTD web-based analysis tools can quickly identify, rank, and prioritize 39 drugs (that are current treatments for neuroblastoma) as candidates for repositioning against B-cell chronic lymphocytic leukemia, with cisplatin and JQ1 as two lead compounds.

Going forward, the extent and content of this dataset will continue to grow with each monthly update at CTD. Importantly, GO terms are also currently used by CTD biocurators to curate phenotypes [6] and exposure outcomes in a new exposure module [45]. Thus, the “GO-to-disease” resource reported here will provide further ways to connect information used by diverse researchers, allowing seamless data links between GO annotations, inferred diseases, phenotypes, and exposure science.

## Supporting Information

S1 File Disease and GO term data for repositioned drugs. Inferred GO-BP terms retrieved for diseases treated by the repositioned drugs raloxifene, thalidomide, sildenafil, and for hypothetical drug against Alzheimer disease and type 2 diabetes.
(XLS)

S2 File Contingency tables. Contingency tables used to determine the significance of overlap for Venn diagrams.
(PDF)

S3 File Inferred GO-MF terms for six cancers. All inferred GO-MF terms for six cancers and the 210 inferred GO-MF terms shared by all six cancers: breast neoplasms, hepatocellular carcinoma, colorectal neoplasms, lung neoplasms, prostatic neoplasms, and stomach neoplasms.
(XLS)

S4 File List of 2,457 disease-pairs and their statistical metrics. The 2,457 disease-pairs with their individual number of genes, number of overlapping genes, number of inferred GO-BP terms, and number of overlapping inferred GO-BP terms. Statistical metrics include similarity indices, unadjusted p-values, and Bonferroni adjusted p-values.
(XLSX)

S5 File Distribution analysis. The distribution of the number of shared inferred GO-BP terms for the 2,457 disease-pairs.
(PDF)
