# Supplementary material for: Generating Gene Ontology-Disease Inferences to Explore Mechanisms of Human Disease at the Comparative Toxicogenomics Database
Source: PLoS One. 2016 May 12;11(5):e0155530. doi: 10.1371/journal.pone.0155530 (PMC4865041; doi:10.1371/journal.pone.0155530)
Supplement: S2 File — Contingency tables used to determine the significance of overlap for Venn diagrams. (PDF) [file pone.0155530.s002.pdf]

Supplemental File S2: Contingency Tables

The following contingency tables were used to determine the significance of overlap for each Venn diagrams in Figures 3, 4, and 6.

For shared inferred GO-BP analyses, the background were the set of all GO BP terms that were inferred from any disease in CTD. For shared direct gene analyses, the background were all genes associated to any disease in CTD. The “phyper” function in R 3.2.1 was used to analyze the following 2x2 contingency tables using the hypergeometric distribution.

The “chi.test” function in R 3.2.1 was used to analyze the overlap among the inferred GO-BP terms for Bipolar Disorder, Chronic Kidney Failure and Diaphragmatic Hernia using Pearson’s Chi-squared test as shown in the last table.

Diseases Analyzed: osteoporosis and breast cancer
Disease A: osteoporosis
Disease B: breast cancer

Shared Inferred GO-BP

	**Disease A**	**Not Disease A**	**Total (Background)**
**Disease B**	929	3,143	4,072
**Not Disease B**	287	6,251	6,538
**Total (Background)**	1,216	9,394	10,610

p-value < 0.001

Shared Direct Genes

	**Disease A**	**Not Disease A**	**Total (Background)**
**Disease B**	9	424	433
**Not Disease B**	57	6,252	6,309
**Total (Background)**	66	6,676	6,742

p-value = 0.009

Diseases Analyzed: leprosy and multiple myeloma
Disease A: leprosy
Disease B: multiple myeloma

Shared Inferred GO-BP

	**Disease A**	**Not Disease A**	**Total (Background)**
**Disease B**	88	697	785
**Not Disease B**	180	9,645	9,825
**Total (Background)**	268	10,342	10,610

p-value < 0.001

Shared Direct Genes

	Disease A	Not Disease A	Total (Background)
**Disease B**	0	31	31
**Not Disease B**	12	6,699	6,711
**Total (Background)**	12	6,730	6,742

p-value = 0.054

Diseases Analyzed: angina pectoris and erectile dysfunction
Disease A: angina pectoris
Disease B: erectile dysfunction

Shared Inferred GO-BP

	Disease A	Not Disease A	Total (Background)
**Disease B**	110	430	540
**Not Disease B**	268	9,802	10,070
**Total (Background)**	378	10,232	10,610

p-value < 0.001

Shared Direct Genes

	Disease A	Not Disease A	Total (Background)
**Disease B**	0	10	10
**Not Disease B**	34	6,698	6,732
**Total (Background)**	34	6,708	6,742

p-value = 0.049

Diseases Analyzed: type 2 diabetes and Alzheimer disease
Disease A: type 2 diabetes
Disease B: Alzheimer disease

Shared Inferred GO-BP

	Disease A	Not Disease A	Total (Background)
**Disease B**	1,108	703	1,811
**Not Disease B**	1,189	7,610	8,799
**Total (Background)**	2,297	8,313	10,610

p-value < 0.001

Shared Direct Genes

	Disease A	Not Disease A	Total (Background)
**Disease B**	11	84	95
**Not Disease B**	122	6,525	6,647
**Total (Background)**	133	6,609	6,742

p-value < 0.001

Diseases Analyzed: ulcerative colitis and coronary artery disease
Disease A: ulcerative colitis
Disease B: coronary artery disease

Shared Inferred GO-BP

	Disease A	Not Disease A	Total (Background)
**Disease B**	398	644	1,042
**Not Disease B**	676	8,892	9,568
**Total (Background)**	1,074	9,536	10,610

p-value < 0.001

Shared Direct Genes

	Disease A	Not Disease A	Total (Background)
**Disease B**	0	60	60
**Not Disease B**	60	6,622	6,682
**Total (Background)**	60	6,682	6,742

p-value = 0.416

Diseases Analyzed: Bipolar Disorder, Chronic Kidney Failure and Diaphragmatic Hernia
Disease A: Bipolar Disorder
Disease B: Chronic Kidney Failure
Disease C: Diaphragmatic Hernia

Shared Inferred GO-BP

	Disease A&B	Disease A & Not Disease B	Disease B & Not Disease A	Not Disease A & Not Disease B	Total (Background)
**Disease C**	231	86	452	627	1,396
**No Disease C**	71	403	512	8,228	9,214
**Total (Background)**	302	489	964	8,855	10,610

Chi-Square = 2314.6, p < 0.001
