# Supplementary material for: Generating Gene Ontology-Disease Inferences to Explore Mechanisms of Human Disease at the Comparative Toxicogenomics Database
Source: PLoS One. 2016 May 12;11(5):e0155530. doi: 10.1371/journal.pone.0155530 (PMC4865041; doi:10.1371/journal.pone.0155530)
Supplement: S5 File — The distribution of the number of shared inferred GO-BP terms for the 2,457 disease-pairs. (PDF) [file pone.0155530.s005.pdf]

Supplemental File S5: Distribution of inferred GO-BP terms for 2,457 disease-pairs

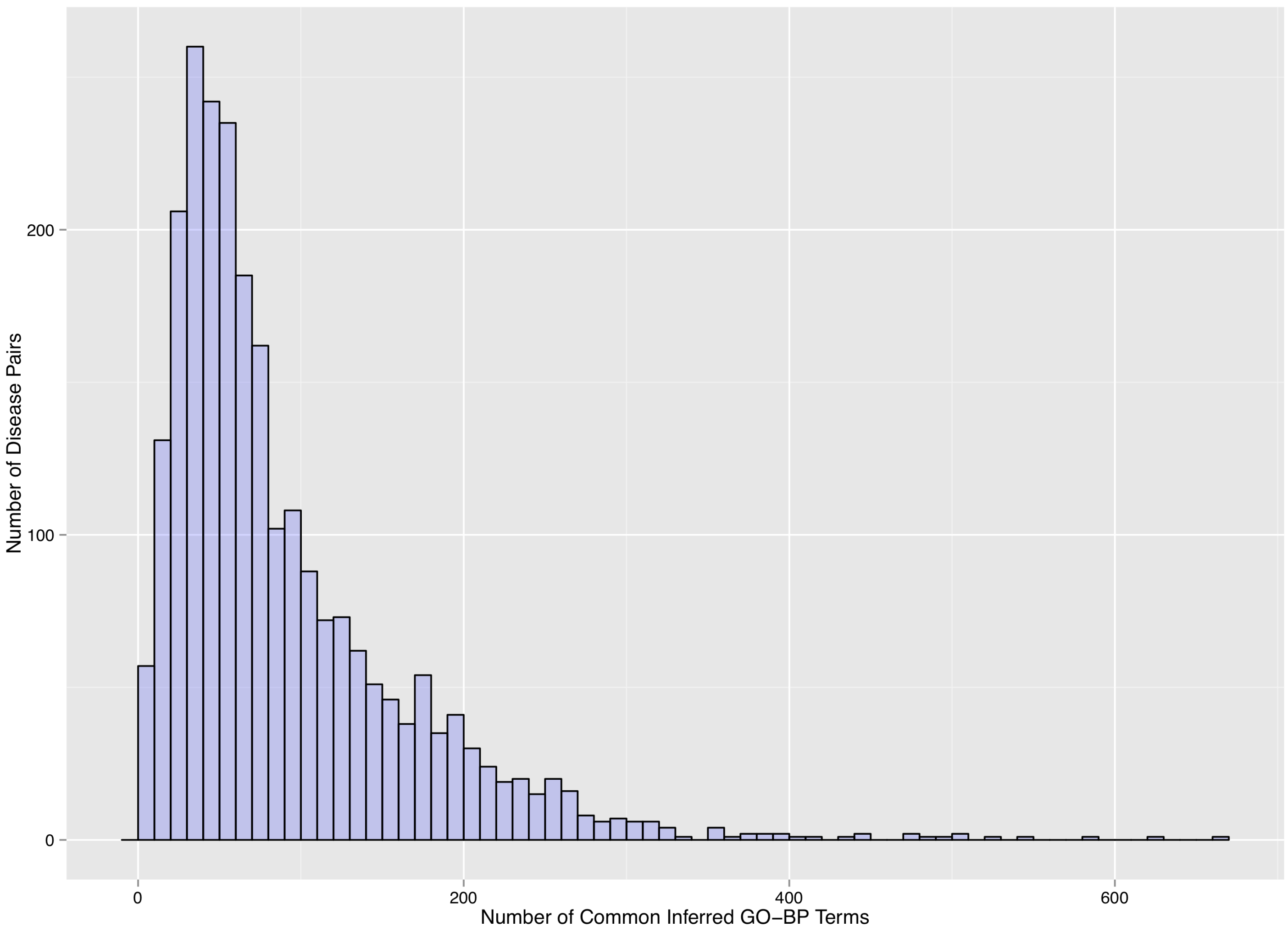

Supplemental Figure 1: The distribution of the number of shared inferred Gene Ontology Biological Process (GO-BP) terms for the 2,457 disease-pairs. The disease-pairs do not share any curated genes and the number of curated genes for each disease in the pair is within 10% of each other. For simplicity, values on the x-axis are bins of 10.

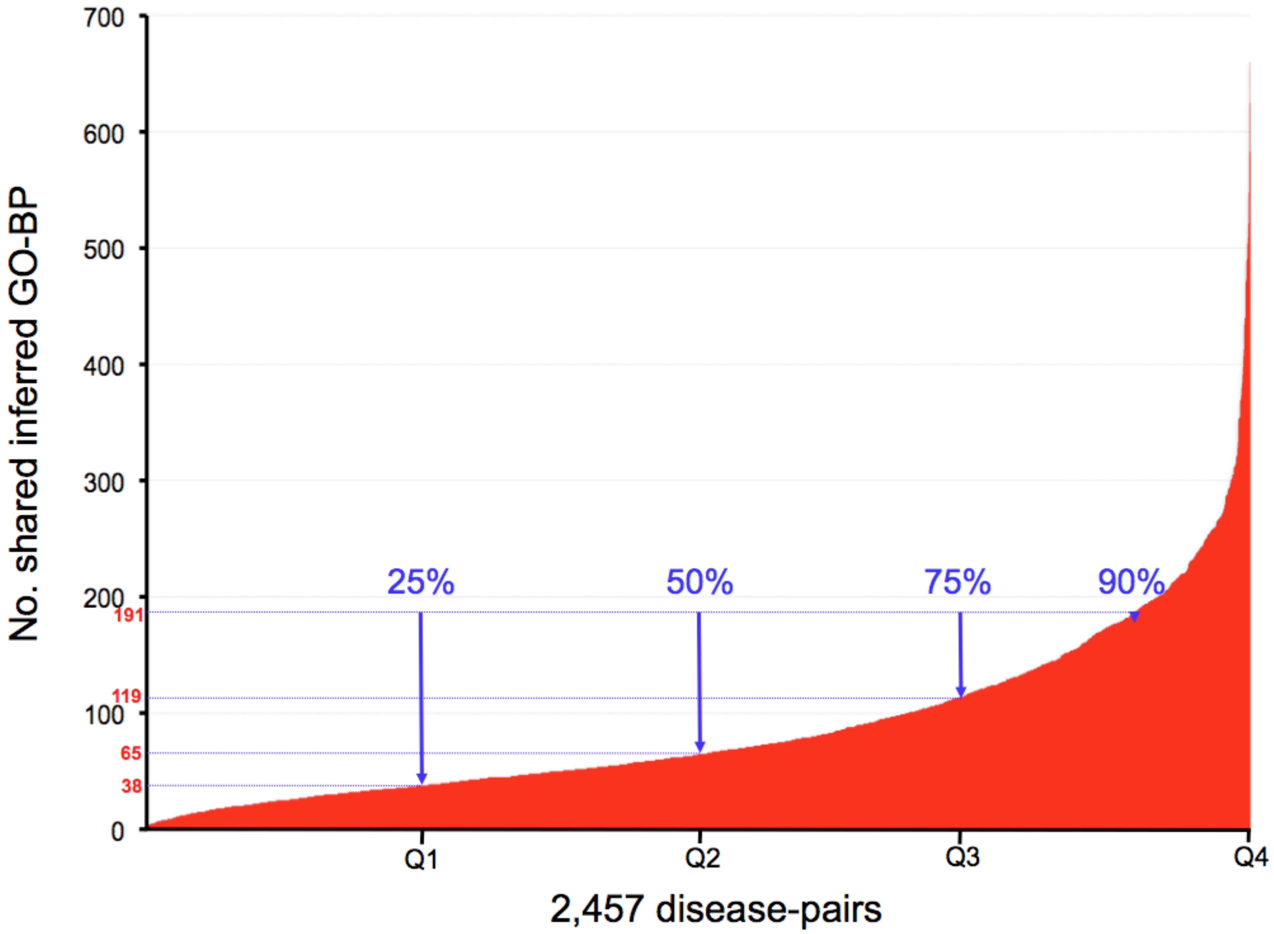

Supplemental Figure 2: The 2,457 disease-pairs binned into quartiles, each containing 614 disease-pairs. The first quartile (Q1) shows that 25% of all disease-pairs have 38 or fewer shared inferred GO-BP terms. The median (Q2) and third quartiles (Q3) are 65 and 119, respectively. Overall, 90% of the disease-pairs have 191 or fewer shared inferred GO-BP terms.
